# A Randomized Controlled Trial Evaluating the Effectiveness of Face-to-Face and Digital Training in Improving Child Mental Health Literacy Rates in Frontline Pediatric Hospital Staff

**DOI:** 10.3389/fpsyt.2020.570125

**Published:** 2021-02-11

**Authors:** Jennifer O'Connell, Roz Shafran, Helen Pote

**Affiliations:** ^1^Department of Psychology, Royal Holloway, University of London, Egham, United Kingdom; ^2^UCL Institute of Child Health, London, United Kingdom

**Keywords:** mental health literacy, randomized controlled trial (RCT), pediatric, digital training, face to face training, frontline staff, recognition, confidence

## Abstract

**Background:** Children with chronic physical health conditions are up to six times more likely to develop a mental health condition than their physically well peers. Frontline pediatric hospital staff are in a good position to identify mental health problems and facilitate appropriate support for patients. To date, no evaluation of mental health literacy training has taken place with this professional group to enable early identification of difficulties. It is also not known whether face-to-face or digital training is more effective or preferable in this setting. To improve the skills of frontline hospital staff, a face-to-face and digital mental health literacy training course was delivered using MindEd content and evaluated in a randomized controlled trial.

**Method:** Two-hundred and three frontline staff across different professions from a tertiary pediatric hospital were randomized to a face-to-face (*n* = 64), digital (*n* = 71), or waitlist control group (*n* = 68). Face-to-face training was two and a half hours and digital training took ~1 h. The effects of training were evaluated pre- and post-training and at two-week follow-up. Questionnaires assessed mental health knowledge, stigma, confidence in recognizing concerns and knowing what to do, actual helping behavior, as well as training delivery preference, completion rate, and satisfaction.

**Results:** Both face-to-face and digital training increased mental health knowledge, confidence in recognizing mental health problems and knowing what to do compared to waitlist controls. Digital training increased actual helping behavior relative to the waitlist controls and stigma decreased across all groups. Staff were satisfied with both delivery methods but preferred face-to-face training.

**Conclusions:** The results provide promising findings that digital content is an effective way of improving mental health literacy in frontline pediatric hospital staff. Providing digital training could be a time-efficient way of upskilling non-mental health professionals to identify mental health needs in a pediatric population and facilitate access to appropriate care.

## Introduction

Mental health problems in children and young people are common but only a minority receive specialist mental health support (1). If children also have a chronic physical health or neurological condition (e.g., diabetes, asthma, epilepsy), the risk of developing a mental health problem can increase by up to six-fold [e.g., (2, 3)]. Young people with chronic illnesses are more likely to have higher levels of internalizing and externalizing problems than their physically healthy peers (4). This can in part be understood in the context of increased stressors such as undergoing rigorous treatment and disease management, lifestyle changes, feelings of isolation, and stigmatization (5). Many pediatric services have dedicated psychology support, however, for referrals made to Child and Adolescent Mental Health Services only a small minority mention chronic illness (6) indicating the need for improved recognition of mental health problems in young people with chronic illness. Such improved recognition would facilitate early intervention and associated benefits in terms of clinical outcomes (7).

Despite the importance of recognition of mental health problems in young people with chronic illness, 42% of practice nurses reported that they had no mental health training at all and 82% reported they felt ill-equipped to deal with aspects of mental health for which they are responsible (8). These findings indicate an urgent need to improve mental health literacy for those working in pediatric settings. The term “mental health literacy” arose from work within health literacy (9) and refers to an individuals' “knowledge and beliefs about mental disorders which aid their recognition, management or prevention” [(10), p. 182].

Current research indicates worryingly low levels of mental health literacy amongst medical staff (11) with hospital staff who have inadequate awareness of mental health being more likely to have stigmatized attitudes to mental health, which may lead to feelings of anxiety among staff and a desire to avoid clients, resulting in poor quality care and less effective outcomes (12).

These reports on hospital staff's confidence and competence within the field of mental health are consistent with the knowledge that young people prefer to speak to a close friend or family member, rather than speak to a professional about their mental health (13). The notion that young people may prefer to speak to their loved ones rather than a professional is consistent with the value of peer support and lay help (14), though it has been shown that young people have problems recognizing symptoms of mental illness but encouragement from others can aid in help-seeking behavior (15). It is therefore essential for adults who are in regular contact with young people to be trained to recognize mental health problems and know how to act to enable them to seek appropriate help (16).

Adult mental health literacy training programs have been extensively evaluated. For example, six randomized controlled trials (RCTs) have found that the 12-h face-to-face “Mental Health First Aid” course [MHFA; (17)] show improvements in knowledge and confidence to provide help to another adult, decreased stigmatized attitudes, and increased helping behavior, with changes being maintained six months post-training. These results have been shown across a variety of different settings internationally including in government workplace settings (18, 19), educational settings (20, 21), with nursing students (22), and across the general public (23).

A recent review of 21 studies of child mental health literacy training programs that have been delivered to non-specialist professionals working with children suggests that global and specific child mental health literacy training improved professionals' knowledge and stigma-related attitudes toward mental health (24). Training content was heterogeneous and tended to reflect the specific needs of the target population. It was highlighted that few studies examined helping actions taken to benefit the people that the training programme ultimately serves and it recommends future studies focus on RCTs with follow-up time periods that address actual helping behavior. The majority of mental health literacy interventions were delivered face to face and a minority were delivered digitally. When given the option to self-select onto a face-to-face or digital course, there appears to be no set preferences among participants or difference in outcomes between groups which is in line with research on digitally delivered and face-to-face interventions (25).

In an uncontrolled study, 37% of nursing and medical students (mean age of 29) opted for the face-to-face course over a digital option (26), whereas the reverse was observed for financial counselors (mean age of 49) with 82% opting for the face-to-face course (27). Satisfaction rates in the latter study demonstrated no difference between delivery method, with 95% and 94% enjoying the digital and face-to-face course, respectively. In both studies, mental health literacy and confidence to provide help were shown to improve, however results must be taken with caution as there was no control group. These findings may reflect different course delivery preferences between professionals and perhaps individuals of certain ages, suggesting that training programmes should not take a “one size fits all” approach.

To date, no RCTs have directly compared face-to-face and digital adult or child mental health literacy trainings. It would be useful to compare these approaches in terms of preference, satisfaction, and completion rates to determine which method might be most acceptable for different professionals and cost efficient. The importance of establishing the efficacy and acceptability of digital training has increased since the global pandemic and the need for remotely delivered training and provision (28).

The aim of the current study was to examine the impact of child mental health literacy training in frontline pediatric hospital staff who have regular contact with young people. Specifically the study aimed (1) To establish baseline levels of mental health literacy in frontline pediatric hospital staff and (2) to compare face-to-face delivery (F2F) with digital delivery (DD) against a waiting list control group condition where no training was provided (CG). It was hypothesized that (1) The DD and F2F will show improvements in mental health knowledge compared to the CG, (2) the DD and F2F will show reduced stigma-related mental health knowledge and behaviors compared to the CG, (3) the DD and F2F will be more confident in recognizing and knowing what to do following training compared to the CG, (4) the DD and F2F will report higher levels of actual helping behavior post-training compared to the CG, and (5) there will be no difference in completion rates, preference, or satisfaction between the DD and F2F.

## Method

### Design

**Figure 1:**
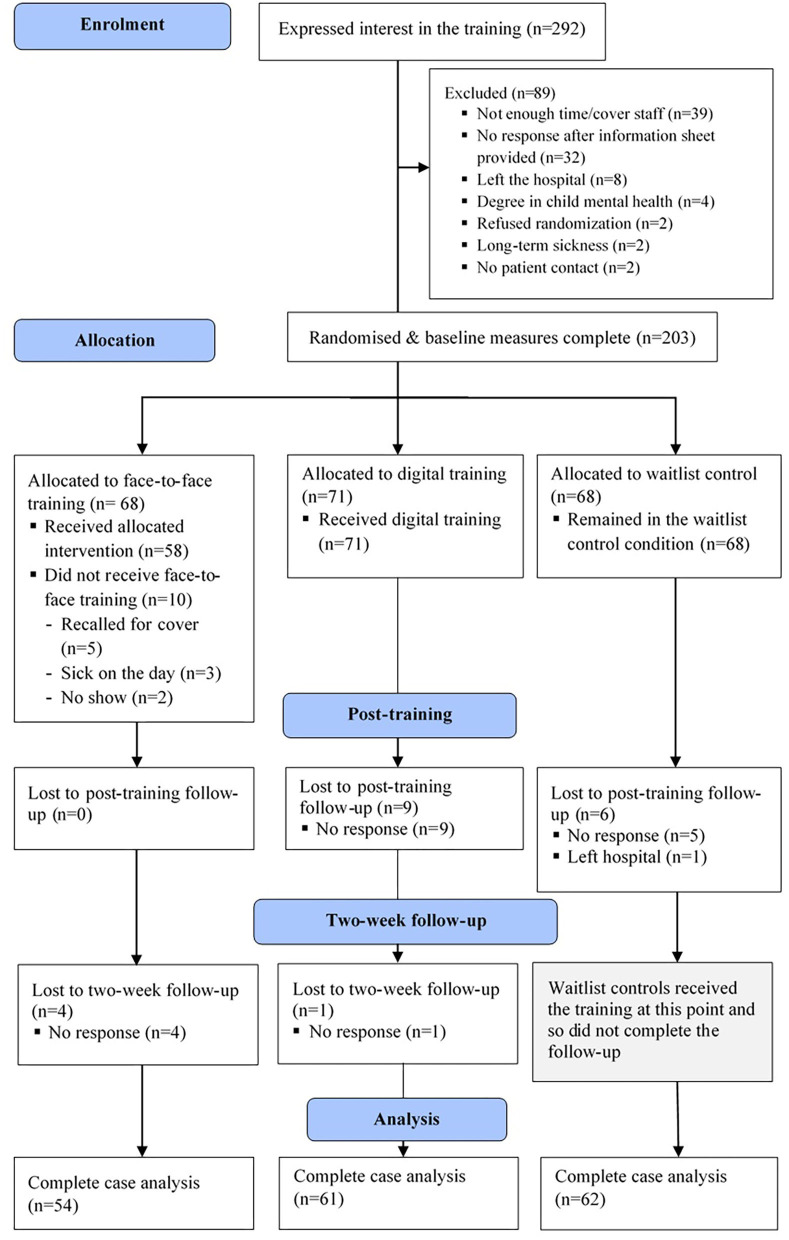
Consort flow diagram.

Frontline pediatric hospital staff were randomized in a three-arm randomized control trial, comparing the mental health literacy of F2F and DD against a CG (see the consort diagram in Figure 1). The data collection points were at pre-training and post-training for all three groups, and there was a two-week follow-up for the intervention groups to ascertain further changes in confidence. Ethical approval for the study was granted by the Health Research Authority (238067) and was approved by Great Ormond Street Hospital Clinical Research Adoptions Committee and Research and Design Department (18PP12). The study was not pre-registered.

### Participants

Two hundred and three frontline pediatric hospital staff were recruited from Great Ormond Street Hospital, London. Participants were recruited through advertisement in the weekly staffing newsletter and through conversations with line managers and discussions in team meetings. Inclusion criteria were: (1) Full, part-time, or honorary employment at the hospital, (2) to be a frontline member of staff, and (3) self-identify as having daily face-to-face contact with patients. Exclusion criteria were: (1) Degree in child mental health, (2) inadequate English to be able to engage with training material and questionnaires, and (3) current participation in a research study or training on child mental health. Participants were randomized to a F2F; (*n* = 64), DD (*n* = 71), or CG (*n* = 68) via a random number generator operated by an independent third party (a graduate student). All participants completed their baseline measures post-randomization online and were then provided access to the training and subsequent questionnaires.

Fifty-eight F2F participants completed the post-training measures (91%), as did 62 of the DD (87%), and 62 (91%) from the CG. At the 2-week follow-up timepoint, 54 F2F (84%) and 61 from the DD participated (86%), and the CG were provided with the digital training. A breakdown of demographic information for each group is summarized in Table 1.

**Table 1:** Participant demographics by group.

	**Study Sample**		
**Demographics**	**Face-to-face (*n* = 64)**	**Digital (*n* = 71)**	**Waitlist controls (*n* = 68)**
**Gender:**			
Female	79.7%	90.1%	83.8%
Male	20.3%	9.9%	16.2%
Mean age (years)	38.9	34.5	37.9
**Ethnic origin:**			
White	65.5%	59.1%	61.8%
Black	14.1%	9.9%	17.7%
Asian	15.7%	23.9%	8.9%
Mixed/Other	4.7%	7%	11.8%
**Religion:**			
Christianity	50%	33.8%	55.9%
Buddhism	0%	1.4%	1.5%
Hinduism	4.7%	1.4%	2.9%
Judaism	3.1%	7%	2.9%
Islam	10.9%	9.9%	7.4%
Other	0%	4.2%	0%
No religion	26.6%	39.4%	27.9%
Prefer not to say	4.7%	4.2%	1.5%
**Mean education (years)**	15.5 (3.1)	16.5 (2.0)	16.1 (2.9)
**Mean working hours (p/w)**	30.7 (15.2)	26.0 (16.5)	22.9 (23.8)
**Mean duration at GOSH (years)**	3.6 (4.4)	3.1 (4.0)	2.5 (3.2)
**Mean number of patients interacted with (p/w)**	41.8 (78.0)	41.5 (89.1)	32.7 (50.4)
**Previous child & adolescent mental health training:**			
Once-off	6.3%	12.7%	7.4%
Multiple *ad-hoc*	1.6%	4.2%	8.8%
Long course	3.1%	1.4%	0%
None	82.8%	73.2%	73.5%
Other	6.3%	8.5%	10.3%

*N = 203. p/w = per week; GOSH = Great Ormond Street Hospital*.

It was a heterogeneous sample of volunteers (*n* = 76), nurses (*n* = 22), security officers (*n* = 16), receptionists (*n* = 14), clinical assistants (*n* = 12), healthcare assistants (*n* = 10), housekeepers (*n* = 10), play workers (*n* = 7), quality and safety officers (*n* = 5), patient liaison officers (*n* = 5), service managers (*n* = 4), teaching staff (*n* = 4), physiotherapists (*n* = 3), speech and language therapists (*n* = 3), staff working on the young people's forum (*n* = 3), chaplains (*n* = 2), data officers (*n* = 2), physiologists (*n* = 2), a press officer (*n* = 1), family support officer (*n* = 1), and a dietician (*n* = 1).

### Power Calculation

Required sample size was estimated using the G-power programme. To detect a medium effect with sufficient power (80%) at the 0.05 significance level, 50 participants were required in each of the groups (29).

### Training Content

#### MindEd Modules

MindEd (www.minded.org.uk) was selected as it is a free educational resource designed by the Department of Health and Department of Education in the United Kingdom for adults to support children and young people's mental health. The training content consisted of two modules, “What Goes Wrong” and “Mind and Body: The Interface.” Additional information was provided about one internalizing mental health condition (depression) and one externalizing condition (oppositional defiant disorder) and what staff can do within their role if they recognize a child who needs support. These conditions were selected as they represented two common mental health conditions that are linked to physical disorders in young people (30). There is no standardized mental health literacy training and so the content selected aligned with the aforementioned Jorm et al. (10) definition of mental health literacy. Within the “What Goes Wrong” module, the content, as described by the MindEd author, allows participants to learn the broad presentations that suggest child or adolescent mental ill-health or vulnerability (e.g., behavioral, emotional and developmental conditions, and mood swings and psychotic thinking) and briefly learn about the types of biological and environmental factors (e.g., genetics, physical illness, school) that can influence the mental health of children and young people and a framework for thinking about these issues.

Within the “Mind and Body: The Interface” module, participants learn how mental health problems can have a negative effect on physical health in children and young people, how mental health problems can be caused by brain disorders, and how physical illness can lead to emotional and behavioral changes in children and make it more likely that they develop mental health problems. They also learned how these joint physical/mental health problems can be helped, the names of available services, and an outline of what the treatment pathway may look like using a case example.

Information on symptoms of oppositional defiant disorder and depression were also included in the training based on selected slides from “The Aggressive/Difficult Child” and “Sad, Bored or Isolated” MindEd modules.

### Measures

Care was taken to ensure that measures were standardized and well-used.

#### Demographics

A demographics questionnaire was completed at baseline.

##### Mental Health Literacy

he Mental Health Literacy Scale [MHLS; (31)] was administered at baseline only to assess the level of mental health literacy among frontline staff in a pediatric hospital so this could be benchmarked against other professions. It was not administered at the post-training or follow-up timepoint as the training content did not match the knowledge section of the questionnaire so no meaningful change in score would be expected. Cronbach's alpha in the current study was 0.61.

##### Knowledge About Mental Health

Changes in mental health knowledge were measured using two vignettes (32, 33) at baseline and post-training. One describes a child presenting with a common externalizing disorder, oppositional defiant disorder, and the other is a teenager presenting with a common internalizing disorder, depression. Participants were asked to (i) identify whether they think the young person has a mental health problem, (ii) rate how concerned they are, (iii) name the identified problem, (iv) list five symptoms that are concerning, (v) suggest three reasons why the individual may be displaying this behavior in a hospital setting, (vi) identify ways of acting on their concerns, and (vii) rate how confident they are on acting on these concerns. Overall knowledge was calculated as a composite of these items. Gender of the young person described in these vignettes was counterbalanced.

##### Stigma

The 12-item Mental Health Knowledge Schedule [MAKS; (34)] was asked at baseline and post-training to measure stigma-related mental health knowledge. Cronbach's alpha in the current study was 0.60.

The 8-item Reported and Intended Behavior Scale [RIBS; (35)] was also completed at baseline and post-training to measure intended future stigmatized behavior. Cronbach's alpha in the current study was 0.80.

##### Confidence Recognizing and Responding to Mental Health Concerns and Actual Helping Behavior

A series of visual analog scales (36) were constructed in order to capture short-term change in confidence levels. All participants at baseline were asked (i) how confident they are in recognizing mental health problems in patients at the hospital, (ii) how confident they are at knowing what to do when they recognize mental health problems in patients, (iii) how many patients they have recognized as having mental health difficulties in the past two weeks, (iv) whether they have reported concerns about a patient to their line manager in the past two weeks, (v) how many times they have reported a concern, and (vi) what their reason for reporting or not reporting was. Post-training, the first and second question were re-asked of the F2F and DD. The CG completed all six questions at their post-training timepoint (two weeks after their baseline questionnaires). At the two-week follow-up timepoint, only the F2F and DD completed the six questions as the CG had now received the online training.

##### Completion Rates, Satisfaction, and Preference

Intervention completion rates were calculated in the F2F by recording participants' attendance. The DD were asked what proportion of the modules they completed post-training.

Feedback regarding satisfaction with teaching was collected post-training using the 12-item Training Satisfaction Rating Scale [TSRS; (37)]. Cronbach's alpha in the current study was 0.95.

Post-training, F2F and DD participants were asked whether they would have preferred to have the training face-to-face, digitally, or had no preference.

### Procedure

Once participants were randomized they were provided a link to complete their baseline questionnaires via the Qualtrics software program. Participants then either arranged a date to complete the face-to-face training, were provided with information on how to access the digital modules, or the “timer” was set for controls to complete the post-training measures in two weeks. Personalized email prompts were sent at one-week intervals for a maximum of four weeks to remind participants to complete their post-training and follow-up questionnaires.

#### Face-to-Face Group

Participants received a two-and-a-half-hour teaching session on the identified MindEd modules followed by 30 min to complete the post-training questionnaires. The teaching session was delivered on 10 separate occasions over a 6-month period to accommodate staff availability, with an average of six attendees *per session*. The F2F completed their post-training questionnaires immediately following training to maximize retention. A follow-up questionnaire was circulated two-weeks post-training.

#### Digital Group

Participants received instructions on how to log onto the MindEd website to access the relevant materials once baseline questionnaires were complete. After two weeks, an email reminder was sent to participants to complete the follow-up questionnaires. Another email was sent to complete the follow-up questionnaire after a further two weeks.

#### Waitlist Control Group

Participants completed their post-training measures two weeks post-baseline. They were then given access to the MindEd modules to review in their own time.

### Analyses

The difference in MHLS mean scores between the current sample and other studies were calculated manually via two-tailed independent samples *t*-tests using the respective mean, standard deviation, and sample size. Mixed between-subjects ANOVAs were used to compare the intervention (F2F or DD) to the CG on mental health knowledge of oppositional defiant disorder, stigma-related knowledge and future intended behavior scores, and confidence in recognizing and knowing what to do about mental health concerns. A series of paired samples *t*-tests were subsequently used to assess if there were changes in confidence within each group over time. Baseline knowledge of depression scores differed between groups so an ANCOVA, controlling for baseline scores, was used to compare the interventions to the CG. Three chi-squared tests for independence were used to assess for a difference between groups on reporting of concerns post-training, training completion rates, and training preference. A one-way between-groups ANOVA assessed for differences in satisfaction rates between intervention groups. All analyses were performed using SPSS 21.

## Results

### Assumptions

All assumptions for the statistical analyses were met.

#### Aim 1: Mental Health Literacy Levels Among Frontline Pediatric Hospital Staff

Frontline staff scored an average of 130.9 (*SD* = 12.7) on the Mental Health Literacy Scale (MHLS), with scores ranging from 85 to 154. This was measured at baseline only to benchmark the scores against other professional groups using independent samples *t*-tests. As one might expect, these results are below that of mental health professionals [M = 145.5, *SD* = 7.2, *N* = 43; (31)], *t*_(106)_ = 10.33, *p* < 0.0001. They were also found to be lower than members of the clergy [M = 134.2, *SD* = 10.8, *N* = 238; (38)], *t*_(414)_ = 3.18, *p* = 0.002 who presumably have some experience of working with individuals with mental health concerns. Frontline pediatric staff's MHLS scores were found to be higher than a community sample [M = 127.38, *SD* = 12.63, *N* = 372; (31)], *t*_(413)_ = 3.18, *p* = 0.001 and UK medical students [M = 127.56, *SD* = 11.8, *N* = 25; (11)], *t*_(417)_ = 2.88, *p* = 0.004 who may not have had any prior exposure to working with mental health concerns. Finally, the baseline MHLS scores were found to be the same as those of UK teachers [M = 129.43, *SD* = 12.01, *N* = 144; (39)], *t*_(318)_ = 1.10, *p* = 0.27, prior to them receiving a digital mental health literacy intervention.

#### Aim 2, Hypothesis 1: The DD and F2F Will Show Improvements in Mental Health Knowledge Compared to the CG

Prior to training, 15.6% of F2F, 29.6% of DD, and 13.2% of CG participants identified Gabriel to be suffering from oppositional defiant disorder while 75% of F2F, 85.9% of DD, and 80.2% of CG participants identified Justine to be suffering from depression. After training, this increased to 89.7% and 89.7% in the F2F, 87.1% and 96.8% in the DD, and 14.3% and 82.3% in the CG, respectively.

**Figure 2:**
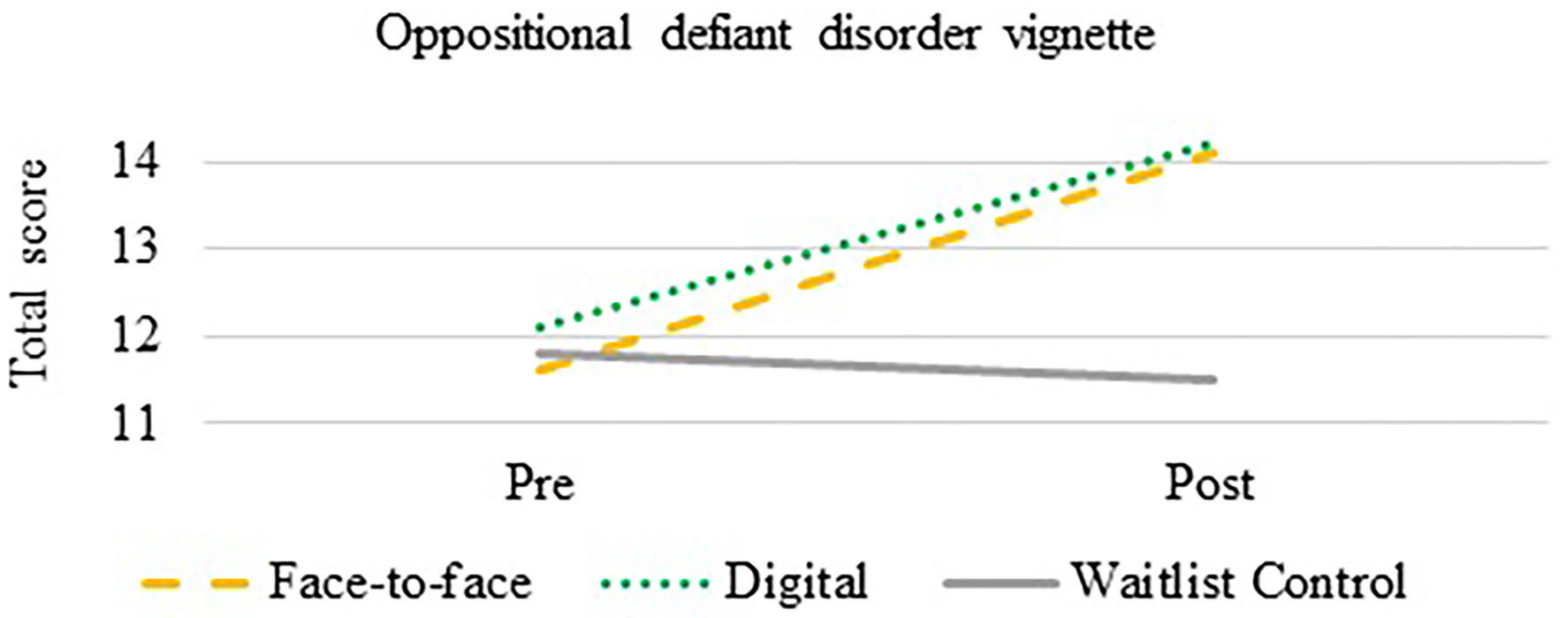
Oppositional defiant disorder mental health knowledge change over time.

A mixed between-within subjects ANOVA was used to compare the effectiveness of the intervention on total mental health knowledge of oppositional defiant disorder. There was a statistically significant effect of time on knowledge [*F*_(1,180)_ = 54.1, *p* < *0.0*001] and an interaction effect between group and time [*F*_(1,180)_ = 19.6, *p* < 0.0001]. Both the F2F (*p* = 0.005, Cohen's *d* = 1.12) and DD (*p* < *0.0*001, Cohen's *d* = 1.13) improved in their overall knowledge of oppositional defiant disorder compared to the CG (see Figure 2).

**Figure 3:**
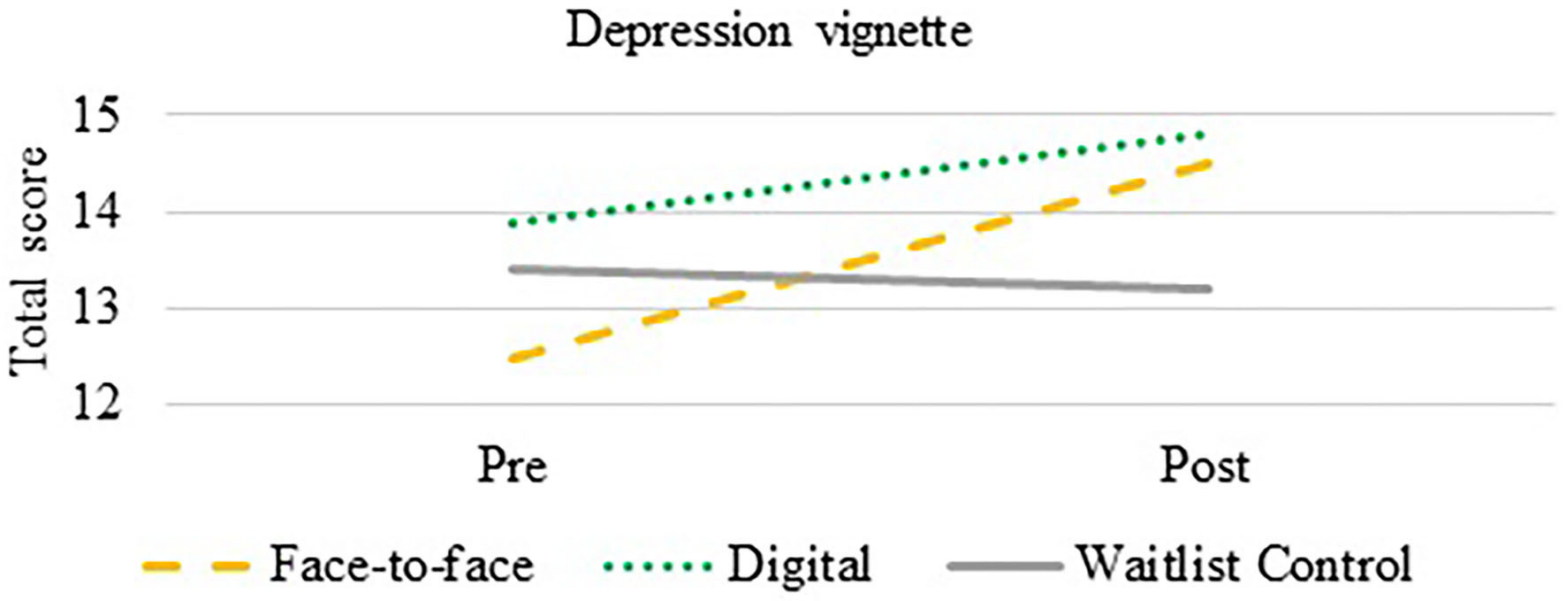
Depression mental health knowledge change over time.

An ANCOVA was used to compare the effectiveness of the intervention on mental health knowledge of depression. After controlling for total pre-training scores, there was a statistically significant difference between groups on post-training total scores over time, *F*_(2,178)_ = 14.76, *p*< *0.0*001. Both the F2F (*p* < 0.0001, Cohen's *d* = 0.53) and DD (*p* < *0.0*001, Cohen's *d* = 0.74) improved their knowledge compared to the CG (see Figure 3).

#### Hypothesis 2: The DD and F2F Will Show Reduced Stigma-Related Mental Health Knowledge and Behaviors Compared to the CG

A mixed between-within subjects ANOVA was conducted to assess the impact of training on stigma-related knowledge on the MAKS and intended future discriminatory behavior toward people with mental illness on the RIBS (see Table 2). In both measures, higher scores indicate reduced stigma.

**Table 2:** Change in mean (SD) stigma scores over time.

	**Face-to-face**			**Digital**			**Waitlist Control**		
	**Pre (*n =* 64)**	**Post (*n =* 58)**	***d***	**Pre (*n =* 71)**	**Post (*n =* 62)**	***d***	**Pre (*n =* 68)**	**Post (*n =* 61)**	***d***
MAKS Total	22.1 (4.5)	25.7 (2.8)	0.98	21.2 (4.2)	24.4 (2.7)	0.93	22.1 (4.2)	24.6 (2.7)	0.72
RIBS Total	14.3 (4.4)	17.1 (3.3)	0.72	15.5 (4.3)	17.9 (2.6)	0.70	15.4 (4.2)	17.5 (2.7)	0.61

*d = Cohen's d*.

On the MAKS, there was a statistically significant main effect of time on stigma-related knowledge scores, *F*_(1,178)_ = 116.6, *p* < 0001, with all three groups showing improved knowledge at the post-training timepoint. There was no significant interaction between group and time, *F*_(2,178)_ = 1.3, *p* = 0.27. The main effect of comparing groups was not significant, *F*_(2,178)_ = 1.8, *p* = 0.16, suggesting that the training delivery method was not a contributing factor to the change in stigma-related mental health scores.

On the RIBS, there was a statistically significant main effect of time on intended future discrimination scores, *F*_(1,178)_ = 95.2, *p* < 0.0001, with all three groups showing reduced stigma at the post-training timepoint. There was no significant interaction between group and time, *F*_(2,178)_ = 0.57, *p* = 0.24, and similar to the MAKS, the main effect of comparing groups was not significant, *F*_(2,178)_ = 1.4, *p* = 0.24, further suggesting that it was not the training delivery method that decreased intended future discrimination scores.

#### Hypothesis 3: The DD and F2F Will Be More Confident in Recognizing and Knowing What to Do Following Training Compared to a CG

Table 3 presents participants' confidence levels with regards to recognizing and knowing what to do when they notice mental health problems in young people.

**Table 3:** Confidence in recognizing and knowing what to do about mental health concerns.

		**Pre (T1)**	**Post (T2)**	**Follow-up (T3)**	***t-*tests**
Face-to-face	Recognizing	4.0 (1.4)	5.4 (1.4)	6.1 (0.6)	T2 > T1^***^ T3 > T1^***^ T3 > T2^***^
	What to do	4.2 (1.5)	5.8 (1.6)	6.3 (0.8)	T2 > T1^***^ T3 > T1^***^ T3 > T2^*^
Digital	Recognizing	4.5 (1.4)	5.1 (1.7)	5.8 (0.9)	T2 > T1^**^ T3 > T1^***^ T3 > T2^*^
	What to do	4.6 (1.4)	5.4 (1.9)	6.2 (1.0)	T2 > T1^*^ T3 > T1^***^ T3 > T2^**^
Control	Recognizing	4.2 (1.4)	4.5 (1.4)	-	*Ns*
	What to do	4.3 (1.5)	4.7 (1.5)	-	*Ns*

*** p < 0.0001;

** p < 0.005;

* *p < 0.05; ns = non-significant*.

### Confidence Recognizing

A mixed between-within subjects ANOVA found that there was a statistically significant main effect of time, *F*_(1,179)_ = 33.7, *p* < 0.0001, on confidence in recognizing mental health problems and an interaction effect between time and group, *F*_(2,179)_ = 7.5, *p* = 0.001. There was no main effect of group, *F*_(2,179)_ = 2.7, *p* = 0.07. There was a significant difference between the F2F and CG (*p* < 0.0001, Cohen's *d* = 0.64) and between the DD and CG (*p* = 0.02, Cohen's *d* = 0.39) on pre- and post-training confidence in recognition of mental health concerns. The lack of comparison with the CG at follow-up is recognized as a limitation of this study.

### Confidence Knowing What to Do

A mixed between-within subjects ANOVA found that there was a statistically significant main effect of time, *F*_(1,179)_ = 41.6, *p* < 0.0001, on confidence in knowing what to do, and an interaction effect between time and group *F*_(2,179)_ = 7.4, *p* = 0.001. There was a main effect of group on confidence with respect to knowing what to do, *F*_(2,179)_ = 3.5, *p* = 0.03. There was a significant difference between the F2F and CG (*p* = 0.03, Cohen's *d* = 0.71) and between the DD and CG (*p* = 0.04, Cohen's *d* = 0.41) on pre- and post-training confidence in knowing what to do when a mental health concern is recognized.

#### Hypothesis 4: The DD and F2F Will Report Higher Levels of Actual Helping Behavior Post-training Compared to the CG

##### Actual Reporting Behavior

With regards to the F2F compared to the CG, a chi-squared test for independence (with Yates Continuity Correction) indicated that there was no significant association between group (F2F and CG) and reporting of mental health concerns (yes or no) prior to training, $\chi_{\left(1,\text{n}=132\right)}^{2}$ = 0.00, *p* = 1.0. There was also no significant association post-training, $\chi_{\left(1,\text{n}=108\right)}^{2}$ = 1.94, *p* = 0.16, suggesting that the increase in the F2F reporting behavior observed in Table 4 did not reach significance.

**Table 4:** Percentage of staff who reported identifying mental health concerns.

**Group**	**Baseline**	**Follow-up**
Face-to-face	9.4%	19.1%
Digital	16.9%	30.4%
Waitlist control	10.3%	8.2%^*^

* *These data were collected at the post-training timepoint*.

With regards to the DD compared to the CG, a chi-squared test for independence indicated that there was no significant association between group and reporting mental health concerns prior to training, $\chi_{\left(1,\text{n}=139\right)}^{2}$ = 0.79, *p* = 0.38. There was however a significant association post-training, $\chi_{\left(1,\text{n}=117\right)}^{2}$ = 8.00, *p* = 0.005, suggesting that DD reported significantly more concerns than the CG post-training.

#### Hypothesis 5: There Will Be No Difference in Completion Rates, Preference, or Satisfaction Between the DD and F2F

Completion, preference, and satisfaction results are reported in Table 5. Chi-squared tests for independence (with Pearson) indicated that there was no significant association between group and completion rates but that there was a significant association between group (F2F and DD) and training preference (F2F, DD, and no preference) with participants preferring to receive face-to-face instead of digital training. Finally, a one-way between-groups ANOVA found that the F2F was more satisfied with the training than the DD, with a higher total TSRS score, and objectives and content, method, and usefulness subscale scores.

**Table 5:** Training completion, preference, and mean satisfaction (SD) rating per intervention group.

	**Face-to-face**	**Digital**	**Chi-squared test of independence/One-way ANOVA**
**Completion rate:**			
All	100% (*n =* 58)	88.7% (*n =* 55)	$\chi_{\left(3\right)}^{2}$ = 7.0
Most	-	8.1% (*n =* 5)	*p = 0.0*7
Part	-	1.6% (*n =* 1)	*n =* 120
None	-	1.6% (*n =* 1)	
**Training preference:**			
Face-to-face	93.1%	64.5%	$\chi_{\left(2\right)}^{2}$ = 14.6
Digital	1.7%	14.5%	*p* = 0.001
No preference	5.3%	21.0%	*n =* 120
**Satisfaction (TSRS):**			
Objectives	13.7 (1.6)	12.2 (2.1)	*F*_(1,119)_ = 20.2 *p* < 0.0001
Method	27.6 (2.8)	22.7 (5.0)	*F*_(1,119)_ = 41.4 *p* < 0.0001
Usefulness	13.9 (1.4)	12.7 (2.5)	*F*_(1,119)_ = 11.0 *p = 0.0*01
Total	55.2 (5.2)	47.7 (8.8)	*F*_(1,119)_ = 31.9 *p* < 0.0001 *d* = 1.04

*d = Cohen's d*.

## Discussion

This is the first known RCT evaluation that has successfully delivered a mental health literacy training across frontline staff in a pediatric hospital setting. The first aim of the study was to benchmark baseline mental health literacy levels against other professional groups and the second aim was to increase mental health literacy levels of frontline pediatric hospital staff using MindEd content that was delivered face-to-face or digitally.

Baseline mental health literacy rates suggested that frontline pediatric staff show lower mental health literacy levels than mental health professional and members of the clergy. Frontline pediatric staff showed higher mental health literacy rates than both a community sample and to UK medical students, who may have less exposure to mental health conditions than the above professionals. Interestingly, the pediatric staff showed similar levels of mental health literacy to teachers. Given the UK government's push for educating all primary and secondary school teachers in mental health awareness (40), this finding suggests that frontline pediatric staff may equally benefit from a child mental health literacy training programme to identify and support young people in receiving the appropriate care.

Results from the intervention showed that both the DD and F2F were successful in increasing mental health knowledge of depression and oppositional defiant disorder compared to the CG. It was observed that baseline knowledge about depression was higher than knowledge of oppositional defiant disorder, something that has been observed in previous literature between depression and other lesser known mental health conditions, like schizophrenia [e.g., (41)]. Overall change in oppositional defiant disorder knowledge scores demonstrated a large effect size for the F2F (*d* = 1.12) and DD (*d* = 1.13) compared to the CG. Changes in overall knowledge of depression showed moderate effect sizes for the F2F (*d* = 0.53) and DD (*d* = 0.74) relative to the CG. The effect sizes within the current study are among the largest reported from a brief training and are on par with less rigorous studies of a longer duration [e.g., (42–44)].

The large change in both constructs of stigma across all groups was an interesting result which may be understood in terms of reactivity to measurement or social desirability effects. It was very encouraging to see improvement in self-reported confidence in both recognizing mental health concerns and knowing what to do when these concerns are recognized in both training groups. This is especially noteworthy given the brief duration of the training and that only one other published RCT has assessed and shown improvement in confidence following a two-day (14 h) training (20). The sustained effects along with the improvement in the proportion of participants who reported concerns to relevant professionals, such as escalating it to a line manager or speaking to a mental health care professional in the hospital, is particularly welcome. Participants who did not act on concerns highlighted that there may need to be a strong emphasis on reassuring staff that they do not need to be in a healthcare role to be able to notice and report concerns, not to assume that someone else will have picked it up, and that it is not their role to determine if mental health is of a certain threshold of severity before raising a concern. On the whole, participants were satisfied with the training across delivery methods but there was higher satisfaction and a significantly stronger preference to receive the training face-to-face.

As with all studies, particularly those conducted in “real-world” settings (45) there are a number of limitations to consider when interpreting the findings. Although a RCT design was implemented, the use of stratification variables such as clinical vs. non-clinical professions, duration of time working in the hospital, and number of patients interacted with may have helped reduce the variance observed in the depression vignette baseline data and to more accurately assess the differences between intervention and control groups on the proportion of mental health concerns that are picked up. The oppositional defiant disorder and depression vignette were selected as they represent one of the most common externalizing and internalizing presentations observed within the hospital. A limitation of this is that staff members did not receive an in-depth training on other common presentations (e.g., anxiety), which could mean these symptoms are not picked up as readily by staff. Selecting the “What Goes Wrong” module was an attempt at addressing this issue as it discusses multiple presentations, but future studies could extend the training to include more presentations and use the other young person vignettes developed by Jorm et al. (33) to assess for change. While there are a number of strengths associated with the MindEd training, these resources are not designed specifically for a pediatric population. It may be the case that identification of mental health problems for non-mental health professionals in the context of a pediatric setting may be more challenging and future work is needed to address this and to develop specific training materials to improve recognition of mental health problems in this population in particular.

The self-reports from participants support the hope that beneficiaries (i.e., young people) did receive helpful support, though the evidence is only indirect. Future training studies would benefit from the collection of objective outcome data, specifically with respect to the impact the training has had on professionals taking appropriate actions when mental health concerns are recognized. This could be in the form of formal referrals made and accepted to mental health teams within the hospital or community support. The same vignette was presented twice and it may have been better to present a parallel form to ensure generalizability and prevent reactivity to measurement or undue attention to information in the media about mental health before answering the second time. As mentioned above, this may also account for the decrease in stigma scores observed within the CG.

It would have been preferable to have collected data on confidence levels in the CG at the follow-up timepoint. This was done to reduce likelihood of attrition as a third data collection point would have meant the CG needed to wait additional time before receiving the training material. Although the trend suggests that controls would not have shown a change in confidence scores at this timepoint, this cannot be inferred.

Now that it has been shown possible to implement an RCT in a pediatric hospital across professionals, future studies would benefit from investigating the long-term benefits of the MindEd training, completing a full battery of measures at each time point and increasing the follow-up time point to assess longevity. Some members of staff had been on annual leave or sick leave in the two weeks post-training, so it is possible that the true benefits of the training with regards to recognizing and acting on concerns were not captured within this short window. It is recommended that mental health literacy training be completed as part of staff induction to ensure all staff have the opportunity to complete the training. There is evidence to suggests that a whole systems approach is more effective in supporting people with mental health problems than targeting individual members of the system (46, 47).

## Conclusion

Overall, this study provides promising findings that a brief training can improve the mental health literacy of frontline pediatric hospital staff whether it is delivered digitally or face-to-face. With a wide variety of modules available to freely access on the MindEd platform, this study shows that there is much promise in the impact that increased mental health literacy levels may have on early identification and support in helping vulnerable children and young people get the mental health treatment that they need.

## Data Availability Statement

The raw data supporting the conclusions of this article will be made available by the authors, without undue reservation.

## Ethics Statement

Studies involving human participants are reviewed and approved by National Health Service Health Research Authority, Sponsor Royal Holloway University, London. IRAS 238067, Date: 21st March 2018. The participants provided their written informed consent to participate in this study.

## Author Contributions

RS, HP, and JO'C conceived and planned the RCT and contributed to the interpretation of the results. JO'C carried out the RCT and training, gathered data, analyzed the results, and took the lead in writing the manuscript. All authors provided critical feedback and helped shape the research, analyses, and manuscript.

## Conflict of Interest

The authors declare that the research was conducted in the absence of any commercial or financial relationships that could be construed as a potential conflict of interest. The reviewer KL declared a past supervisory role with one of the authors HP to the handling editor.
